# What matters to women and healthcare providers in relation to interventions for the prevention of postpartum haemorrhage: A qualitative systematic review

**DOI:** 10.1371/journal.pone.0215919

**Published:** 2019-05-08

**Authors:** Kenneth Finlayson, Soo Downe, Joshua P. Vogel, Olufemi T. Oladapo

**Affiliations:** 1 University of Central Lancashire, Research in Childbirth and Health (ReaCH) Group, Preston, Lancashire, United Kingdom; 2 UNDP/UNFPA/UNICEF/WHO/World Bank Special Programme of Research, Development and Research Training in Human Reproduction (HRP), Department of Reproductive Health and Research, World Health Organization, Geneva, Switzerland; 3 Maternal and Child Health Program, Burnet Institute, Melbourne, Australia; National Institute of Health, ITALY

## Abstract

**Background:**

Postpartum haemorrhage (PPH) is a leading cause of maternal mortality and morbidity. Reducing deaths from PPH is a global challenge. The voices of women and healthcare providers have been missing from the debate around best practices for PPH prevention. The aim of this review was to identify, appraise and synthesize available evidence about the views and experiences of women and healthcare providers on interventions to prevent PPH.

**Methods:**

We searched eight electronic databases and reference lists of eligible studies published between 1996 and 2018, reporting qualitative data on views and experiences of PPH in general, and of any specific preventative intervention(s). Authors’ findings were extracted and synthesised using meta-ethnographic techniques. Confidence in the quality, coherence, relevance and adequacy of data underpinning the resulting themes was assessed using GRADE-CERQual. A line of argument synthesis was developed.

**Results:**

Thirty-five studies from 29 countries met our inclusion criteria. Our results indicate that women and healthcare providers recognise the dangers of severe blood loss in the perinatal and postpartum period, but don’t always share the same beliefs about the causes and consequences of PPH. Skilled birth attendants and traditional birth attendants (TBA’s) want to prevent PPH but may lack the required resources and training. Women generally appreciate PPH prevention strategies, especially where their individual needs, beliefs and values are taken into account. Women and healthcare providers also recognize the value of using uterotonics (medications that contract the uterus) to prevent PPH but highlight safety concerns and potential misuse of the drugs as acceptability and implementation issues.

**Conclusions:**

Based on stakeholder views and experiences, PPH prevention strategies are more likely to be successful where all stakeholders agree on the causes and consequences of severe postpartum blood loss, especially in the context of sufficient resources and effective implementation by competent, suitably trained providers.

## Introduction

An estimated 303,000 maternal deaths occurred in 2015 [1]. Recent figures would suggest that more than a quarter of these deaths were due to haemorrhage, with post-partum haemorrhage accounting for almost 20% of all direct deaths [2]. The vast majority of these fatalities took place in low and middle-income settings (LMICs) where more than 200 woman die every hour from a PPH [3]. Despite concerted efforts to reduce these levels of mortality, the issue remains a global challenge.

Primary PPH is commonly defined as a blood loss of 500 ml or more within 24 hours after birth, and, by this definition, approximately 14 million cases of postpartum haemorrhage (PPH) occur each year [2], affecting about 6% of all women giving birth around the world [4,5]. However, in some countries where maternal populations are generally well nourished, unlikely to be anaemic, and where grand multiparity is uncommon, the functional definition is often set at 1000 mls or more [6]. Women who are healthy and not anaemic in pregnancy will recover over time from a blood loss of up to around a litre, but those who are very anaemic, ill, and/or generally in poor health may experience long-term adverse consequences with an observed loss of less than 500mls. There is debate about the accuracy of blood loss measurement in maternity care settings [7], which complicates the assessment of the effectiveness of preventative techniques. While uterine atony is the most common cause of haemorrhage, other factors such as genital tract trauma (i.e vaginal or cervical lacerations), uterine rupture, retained placental tissue, or maternal bleeding disorders can also cause excessive bleeding in the postpartum period [8]. Some clinical characteristics are associated with an increased risk of PPH, including grandmultiparity, prolonged labour and multiple gestation, but the majority of women who experience PPH do not have an antecedent risk factor [9]. Individual risk factors are therefore poor predictors of PPH occurrence [10] and the appropriate care practices to reduce the risk of PPH are not well defined or carried out effectively, particularly in low-income settings [11,12].

Conventional practices to prevent PPH include Active Management of the Third Stage of Labour (AMTSL), although the WHO don’t advocate all of the components of this approach in all settings [13]. This involves prophylactic administration of uterotonic medicines before delivery of the placenta in addition to other non-pharmacological interventions, such as late cord clamping and controlled cord traction of the umbilical cord (in settings where skilled birth attendants are available). Since 2012, WHO has recommended oxytocin (in injectable form) as the uterotonic of choice, or, in the absence of oxytocin, an alternative injectable uterotonic or oral misoprostol (depending on the setting). Although several uterotonic agents have been shown to be effective in preventing PPH, a growing body of literature has highlighted that in many settings, available uterotonics (particularly heat-sensitive agents like oxytocin) may be of poor-quality, which can impair their clinical effects. Uterotonic agents may also have side effects that are uncomfortable for women—for example, women may experience shivering, fever or diarrhoea after taking misoprostol [14,15,16]. This may be distressing in the early hours following birth, when women are trying to form a relationship with their new baby. From a provider perspective there may also be a reluctance to practice routine AMTSL because of concerns about effectiveness and safety. In high-income settings where rapid access to interventions to address an unexpected PPH are available, providers may consider routine PPH prophylaxis unnecessary [17].

In order to optimise guideline implementation by healthcare providers, and their acceptability by service users, it is necessary to establish what matters to these key stakeholders in relation to their views and experiences of postpartum haemorrhage, and the means used to prevent it. No formal appraisal of the qualitative evidence in this field has been conducted to date. The aim of this review is to identify, appraise and synthesize evidence relating to the views and experiences of women and healthcare providers regarding third stage of labour practices for the prevention of PPH. This evidence will be used to inform the updating of WHO recommendations on the prevention and treatment of PPH.

## Methods

We conducted a systematic qualitative review in accordance with the PRISMA guidelines (See S1 Table—PRISMA Checklist). Meta-ethnographic techniques [18] were used for analysis and synthesis. Study assessment included the use of a validated quality appraisal tool [19] and the findings were evaluated for confidence using the GRADE-CERQual tool [20].

### Search strategy

As this is a review of qualitative studies the PICO (Population, Intervention, Control, Outcome) structure was modified to incorporate key subject headings, using the PEO (population, Exposure, Outcome) structure:-

#### Populations

- Women of any parity (including nulliparous) who gave birth in any setting (including in a health facility or at home).
- Birth attendants who care for women during childbirth including healthcare providers, nurses, midwives, doctors, skilled birth attendants, lay health workers, community health workers or traditional birth attendants (see search terms in S1 Appendix for full list).

#### Exposure

- Third stage of labour including a range of interventions and procedures performed for the prevention and treatment of post-partum haemorrhage

#### Outcome

- The views, experiences and perceptions of the populations under investigation with particular regard to the acceptability and feasibility of approaches relating to clinical practices for PPH prevention.

The search terms were organized into broad strings to cover the PEO items outlined above as well as a search string to cover qualitative methodologies. An example of the search strategy is shown in S1 Appendix. In order to achieve an optimal number of studies eight relevant databases were searched, Medline [OVID]; CINAHL [EBSCO]; PsycINFO [EBSCO]; EMBASE [OVID]; Global Index Medicus; LILACS (for studies conducted in South America); AJOL (for studies conducted in Africa) and POPLINE. The searches were conducted on 9^th^ and 10^th^ July 2018.

Where possible, research limiters were used to ensure that searches for qualitative studies were optimized. For example, the research limiter for MEDLINE located ‘Qualitative studies–best balance of specificity and sensitivity’. We searched the reference lists of all eligible studies and used back chaining to locate any relevant studies that were not identified in the electronic searches.

The review was used to inform the updating of WHO’s recommendations on prevention and treatment of PPH, so our searches also included terms relating to the treatment of PPH (e.g. balloon tamponade, NASG, etc;). This paper reports on those studies focusing on the prevention of PPH and a subsequent paper will report on the views and experiences of relevant stakeholders with regard to the treatment of PPH.

### Inclusion/Exclusion criteria

No language restrictions were imposed. The abstracts of studies published in languages other than English were translated into English using freely available online software [Google Translate]. After abstract screening, studies deemed to be relevant were formally translated by colleagues at WHO who were fluent in the selected language. Studies published before 1996 were excluded to ensure that the data reflected the current generation of women and healthcare providers. Case studies, conference abstracts and unpublished PhD or Masters theses were not included.

All studies utilising a qualitative research design (e.g. ethnography, phenomenology), or qualitative methods for data collection (e.g. focus group interviews, individual interviews, observation, diaries, oral histories), and which used qualitative methods for data analysis (e.g. thematic analysis, framework approach, grounded theory, thematic network analysis) were eligible for inclusion. Studies using mixed methods designs were also eligible where it was possible to extract findings derived from the qualitative component. Studies in which data were collected using qualitative methods, but which did not perform a qualitative analysis (e.g. if qualitative data were only reported using descriptive statistics), were excluded.

#### Intervention(s)

We were interested in the prevention of PPH from a qualitative perspective so were looking for any qualitative studies that explored this phenomenon from the perspective of women and healthcare providers. Preventative approaches included the use of uterotonics (oxytocin, carbetocin, ergot alkaloids, ergotamine, ergometrine, oxytocin-ergometrine, misoprostol or prostaglandin) or any associated procedures or practices that influenced the prevention of PPH (active management, uterine massage, uterine packing, uterine compression, cord clamping, controlled cord traction, retained placenta, placental delivery).

#### Study selection

We collated records identified from the different sources into a data management programme (RefWorks) and removed any duplicates. One review author (KF) assessed each abstract in order to determine eligibility for inclusion against the *a priori* inclusion/exclusion criteria. At this stage we removed any abstracts that were clearly irrelevant to the focus of the review. Two review authors (KF, SD) then retrieved the full texts of all the papers that were likely to be relevant, and assessed them independently for eligibility, before agreeing on the final list of included studies. In the event of lack of consensus over the inclusion of a particular study, a third review author (OTO) was available to adjudicate.

We recorded study characteristics using a form designed specifically for this review. The form includes details of: study author, date of publication, country of study, context (urban/rural), region (Africa, Americas, South-East Asian, European, Eastern Mediterranean, Western Pacific), participant group (women or healthcare providers), theory and method used, sample size and quality grading.

#### Quality assessment

The included studies were subject to quality appraisal using an instrument developed by Walsh and Downe [19] and modified by Downe et al [21]. This is a simple appraisal system based on an amalgamation of several published qualitative appraisal tools including the Critical Appraisal Skills Programme (CASP). The system rates studies against 11 pre-defined criteria, and then allocates a score from A-D (see below). Studies scoring C+ or higher will be included in the final analysis.

Scoring criteria for quality appraisal

A: No, or few flaws. The study credibility, transferability, dependability and confirmability are high;

B: Some flaws, unlikely to affect the credibility, transferability, dependability and/or confirmability of the study;

C: Some flaws that may affect the credibility, transferability, dependability and/or confirmability of the study.

D: Significant flaws that are very likely to affect the credibility, transferability, dependability and/or confirmability of the study.

#### Data extraction and analysis

Data extraction and analysis were conducted simultaneously in four steps using methods aligned to meta-ethnography [18] to produce descriptive Summaries of Findings (SoFs–second order constructs). The Summary of Findings statements were evaluated for confidence using the GRADE-CERQual tool (Grading of Recommendations, Assessment, Development and Evaluation)-(Confidence in the Evidence from Reviews of Qualitative Research) [20]. They were then synthesised into third order themes as a basis for deriving a line of argument synthesis from the data.

In step one, basic details of the included papers (author, date, title, country) were indexed and organized into two categories–those representing the views and experiences of women and those representing the views and experiences of health care professionals. For each category the papers were examined, and an index paper selected, chosen to best reflect the focus of the review. The themes and findings identified by the authors of this paper were entered onto a spreadsheet. This process was then continued for all the remaining papers, one at a time, iteratively building a set of themes that captured the quote material presented by the original authors, and their themes and statements, forming the ‘first order constructs’ of this stage of the review.

In step two the first order constructs were refined and merged to form second order constructs (Summary of Findings [SoF] statements), at one remove from the actual data in the included studies. This process includes looking for what is similar between papers (‘reciprocal analysis’), and for what contradicted the emerging findings (‘refutational analysis’). The disconfirming data identified in the on-going refutational analysis were used to refine the emerging constructs, so that the final analysis had high explanatory power for all the data. The second order constructs were developed by KF and agreed by consensus between KF and SD.

In step three, the second order constructs (the SoFs) were assessed for confidence using the GRADE-CERQual tool. This is a recently developed instrument, derived from the approach used in quantitative effectiveness reviews (GRADE). CERQual’s assessment of confidence for individual review findings from qualitative evidence syntheses is based on four components: the methodological limitations of the qualitative studies contributing to a review finding, the relevance to the review question of the studies contributing to a review finding, the coherence of the review finding, and the adequacy of data supporting a review finding. Based on these criteria, review findings were graded for confidence using a classification system ranging from ‘high’ to ‘moderate’ to ‘low’ to ‘very low’. As with study selection, the grades for each review finding were agreed by consensus and where there was disagreement a third reviewer (OTO) was asked to arbitrate.

In step four, the graded review findings were collapsed into over-arching interpretive themes as a means of synthesizing the data into an underlying ‘line of argument’ that describes the whole data set succinctly.

## Results

**Fig 1:**
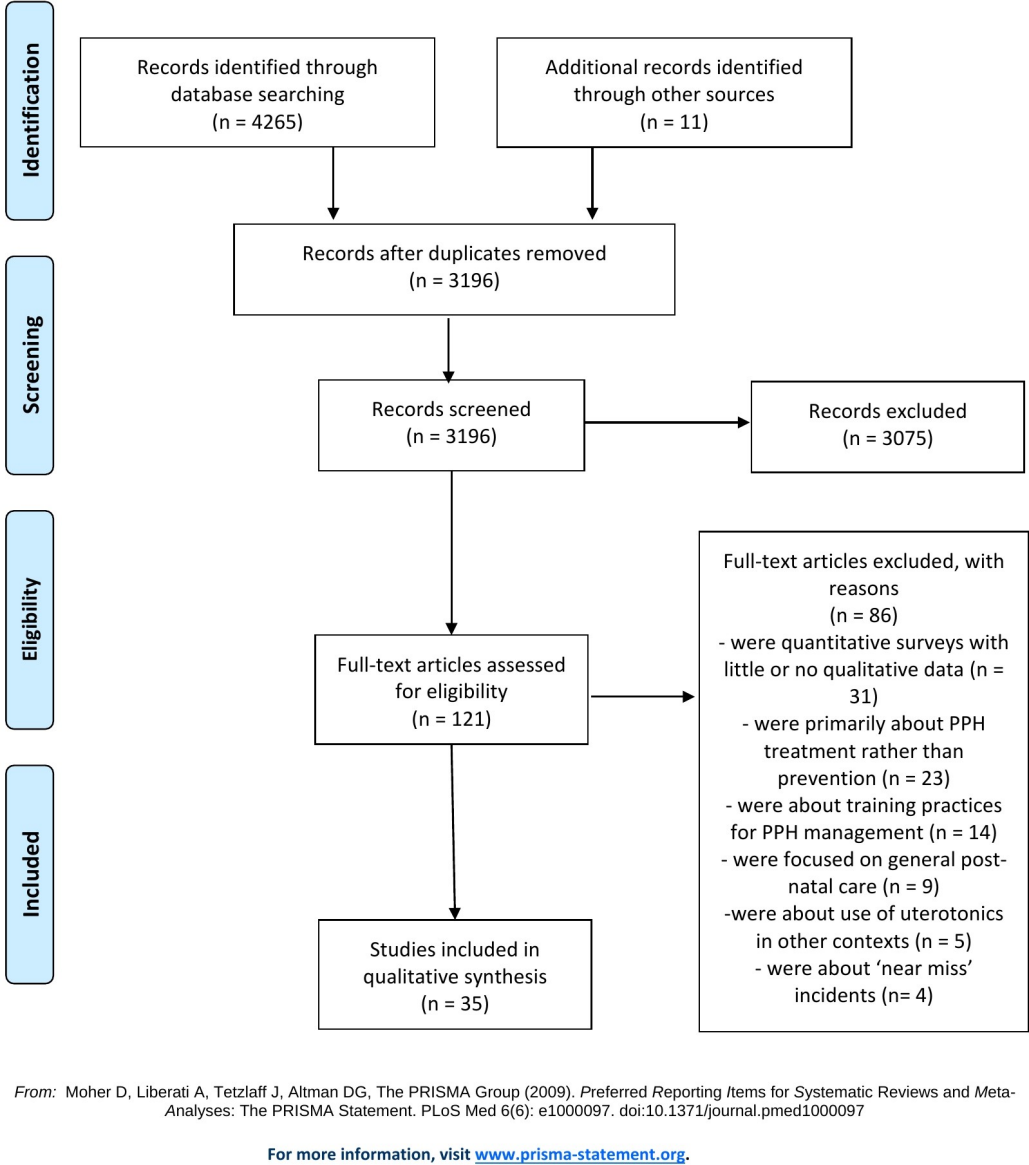
PRISMA flowchart.

Our electronic searches yielded 4265 citations. We screened 3196 unique records after duplicate removal. We assessed 121 full-text articles for eligibility and included 35 studies in this qualitative evidence synthesis. 21 studies reported on the views of healthcare providers, 14 reported on the views of women and 3 reported on both. [See Fig 1].

One study was published in French and, following translation, was excluded on the basis that it contained no pertinent qualitative data [22]. Five studies were about women’s experiences of PPH and were included on the basis that they contained some relevant information about PPH prevention. The included studies, published between 1997 and 2017, were from 29 different countries representing 5 global regions including Europe, South America, Africa, South-East Asia and Australia/New Zealand. The majority of the studies came from LMIC’s (22 in total) with 9 conducted in HIC’s. The studies were conducted in a variety of settings including urban and rural locations and sample sizes ranged from 8 to more than 170 participants [23–57].

Quality assessment of studies ranged from A- to C with an average grade of B. Table 1 lists the study characteristics including the quality assessment grades assigned. Eighteen studies were assessed as having no, few, or only some flaws, with credibility, transferability, dependability, and confirmability unlikely to have been affected (A-B).

**Table 1:** Study characteristics.

Study Number	Authors and Ref	Date	Country	Resource	Participants	Context	Theory and Method	Sample	Quality Rating
1	Álvarez-Franco [23]	2013	Colombia (Americas)	Upper Middle	Women	Urban	Phenomenology with multiple interviews and observations	8 women	C+
2	Dunning [24]	2016	UK (European)	High	Women	Urban	Phenomenology with interviews and observations	11 women (and 6 partners)	B+
3	Robertson [25]	2017	Canada (Americas)	High	Women	Urban	Based on an online survey AND focus groups	8 women	C+
4	Snowdon [26]	2012	UK (European)	High	Women & partners	Urban	Phenomenology including interviews with women and partners	9 women (and 6 partners)	B
5	Thompson [27](a)	2011	New Zealand & Australia (Western Pacific)	High	Women	Urban	Prospective mixed methods study utilizing survey data plus qualitative analysis of narrative data.	171 women (completed the initial survey)	B
6	Kalim [28]	2009	Bangladesh (South-East Asian)	Lower Middle	Women	Rural	Based on surveys and case studies plus interviews with women	38 women plus mother-in-laws	C+
7	Ononge [29]	2016	Uganda (African)	Low	Women & TBA's	Rural	Phenomenology utilizing in depth interviews with women and TBA's	15 women and 6 TBA's.	A-
8	Sibley [30]	2007	Bangladesh (South-East Asian)	Lower Middle	Women & TBA's	Rural	Survey based with semi-structured interview questions delivered verbally	80 women (plus TBA's)	C+
9	Jangsten [31]	2010	Angola (African)	Lower Middle	Women	Urban	Qualitative analysis of open ended questions on a survey with women	102 women	C
10	Matsuyama [32]	2008	Nepal (South-East Asian)	Low	Women, Mothers-in-law & Husbands	Urban & Rural	Grounded theory approach supplemented by case histories	28 participants	B
11	Asowa-Omorodion [33]	1997	Nigeria (African)	Lower Middle	Women	Rural	Anthropological investigation using focus groups with women	20 focus groups with 8–12 women in each	C
12	Fikree [34]	2004	Pakistan (South East Asian)	Lower Middle	Women & TBA's	Urban	Mixed-methods approach utilizing a survey, focus groups and in-depth interviews with women and TBA's	5 focus groups with 8–10 participants plus 15 in depth interviews.	C+
13	Thompson [35](b)	2010	Australia (Western Pacific)	High	Women	Urban	Prospective mixed methods study utilizing surveys plus qualitative analysis of narrative data.	171 women (completed the initial survey)	B
14	Sacks [36]	2017	Uganda & Zambia (African)	Low/Lower Middle	Women	Rural	Evaluation as part of a larger initiative to increase facility birth	48 focus groups including 393 women	C
15	Begley [37]	2012	New Zealand & Ireland (Western Pacific & European)	High	Midwives	Urban	Qualitative descriptive using in-depth interviews and a focus group	27 midwives (18 from NZ and 9 from Ire)	A-
16	Jangsten [38]	2010	Sweden (European)	High	Midwives	Urban	Qualitative descriptive informed by focus groups	32 midwives in 8 focus groups with 3–8 participants in each.	B
17	Kanikasamy [39]	2007	UK (European)	High	Midwives	Urban	Mixed-method approach with surveys	10 midwives	C
18	Schack [40]	2014	Ghana (African)	Lower Middle	Midwives	Urban	Qualitative descriptive informed by individual interviews	12 midwives	A-
19	Deepak [41]	2013	India (South East Asian)	Lower Middle	Providers & Women and mother-in-laws	Urban & Rural	Qualitative descriptive informed by individual interviews	140 interviews with a variety of stakeholders	B-
20	Bazzano [42]	2014	Cambodia (South East Asian)	Lower Middle	Providers (managerial)	National and local policy level providers	Qualitative individual interviews and analysis of government and NGO reports	21 stakeholders	C
21	Beltman [43]	2013	Malawi (African)	Low	Healthcare Workers	Rural	Qualitative descriptive using focus group discussions with pertinent stakeholders	29 stakeholders (8 clinical officers, 14 nurse-midwives and 7 medical assistants)	C+
22	bij de Vaate [44]	2002	Gambia (African)	Low	TBA's (Trained)	Rural	Qualitative descriptive using interviews and focus groups in an iterative, reflective manner	22 TBA's focus groups with 6–10 participants	B-
23	Braddick [45]	2016	Uganda (African)	Low	Healthcare Professionals	Urban & Rural	Observational data and information from interviews with healthcare professionals	18 participants including 4 doctors and 14 midwives (3 from the community settings)	B
24	Garcia [46]	2012	Guatemala (Americas)	Upper Middle	TBA's	Rural	Qualitative descriptive study with 1 focus group	13 TBA's (midwives)	B+
25	Collins [47]	2016	Madagascar (African)	Low	Matrones, Midwives & Physicians	Urban & Rural -	Qualitative descriptive interviews with a variety of different healthcare workers	12 interviews with various providers. Plants also collected to assess uterotonic properties	A-
26	Ith [48]	2013	Cambodia (South East Asian)	Lower Middle	SBA's	Urban & rural	Qualitative descriptive informed by interviews and focus groups	25 SBA's via interviews and focus groups	A-
27	Natarajan [49]	2016	Sierra Leone (African)	Low	Providers	Urban	Evaluation of a PPH training package utilizing surveys and qualitative interviews with key stakeholders	134 providers completed the survey and (x) of these were interviewed	C
28	Ngunyulu [50]	2015	South Africa (African)	Upper Middle	TBA's	Rural	Qualitative exploratory design to compare TBA practices of PN care (from interview data) with 'Western' practices derived from a literature review	15 interviews with TBA's	C+
29	Radoff [51]	2013	Guatemala (Americas)	Upper Middle	TBA's (and auxiliary nurses)	Rural -	Qualitative descriptive with focus groups with population of interest	5 FGD's with 30 TBA's and 9 AN's	B-
30	Sanghvi [52]	2004	Indonesia (South East Asian)	Lower Middle	Community midwives, TBA's and CHW's (and women)	Rural -	Non-randomized experimental design with qualitative interviews to inform acceptability/feasibility aspects	70 providers including 2 FGD's with m/w and 21 interviews with m/w, TBA's or com/ workers	C+
31	Than [53]	2017	Myanmar (South East Asian)	Lower Middle	Midwives, auxiliary midwives and community members	Rural	Qualitative descriptive interviews and focus groups with a variety of providers and community members	15 m/w and 33 AMWs 2 and 5 FGDs respectively plus 36 community reps participated in the FGDs,	B
32	Woiski [54]	2015	Netherlands (European)	High	Healthcare professionals (and women)	Urban	Qualitative descriptive with interviews and focus groups	41 different health professionals (9 Obs, 8 Obs in training, 15 midwives & 9 Obs Nurses) in 4 FGD's	B+
33	Atukunda [55]	2015	Uganda (African)	Low	Government Officials, CSO Representatives and secondary healthcare managers	National—within the context of policy development	Project evaluation looking at availability and distribution of medicines in Africa using interviews with key stakeholders.	82 interviews with a range of officials, representatives and providers. Limited procedural details given	B-
34	Durham [56]	2016	Lao PDR (South East Asian)	Lower Middle	Government Officials, CSO’s, healthcare managers and healthcare professionals	Urban & rural	Qualitative exploratory with interviews supplemented by quantitative 'frequency of response' data	35 interviews using a semi-structured format	B+
35	Spangler [57]	2014	Ethiopia (African)	Low	Government Officials, CSO’s, healthcare managers and healthcare professionals	Regional	Qualitative evaluation of an intervention at national, regional and local levels	42 semi-structured interviews with relevant officials	B

22 Summary of Findings (SoF) statements were derived, including 5 that were shared by women and healthcare providers. CERQual gradings of the review findings ranged from high to very low, with the majority receiving a ‘moderate’ grade for confidence (See S2 Appendix for details of study selection and CERQual Gradings). The SoF statements were synthesised into four interpretive themes. The themes are described below, supported by the SoF’s, illustrated by relevant quotes, and culminating in a synthesis expressed as a Line of Argument (see Table 2 for development of themes).

**Table 2:** Development of themes and line of argument synthesis.

Initial Themes (1^st^ Order)	Summary Finding (2^nd^ Order)	Organizational Theme (3^rd^ Order)	Line of argument synthesis
*Understanding of blood loss [28,29,32–34]*	**Understanding of blood loss.** **Traditional means of preventing or treating PPH.** *Knowledge of complications*. *Desire for more information about PPH*. **Impact of PPH.** Provider preferences for PPH prevention. Desire for more staff training in PPH management. .	What PPH means to stakeholders: beliefs, knowledge, and understanding.	Women giving birth and healthcare providers recognise that severe, uncontrollable blood loss in the peri- and postpartum period is life threatening. Based on their beliefs and experiences, both trained and untrained birth attendants can identify women at risk of PPH, and most are willing to use specific skills and techniques (and, where available, treatments and uterotonic drugs) to reduce the risk. Women generally appreciate this, especially where it is based on their individual needs, beliefs and values. However, there is variation within and between healthcare providers and women, across income settings, in their views as to how far these interventions should be used routinely. In all income settings, systems for PPH prevention are likely to be more successful locally where there is a common frame of reference for the causes and consequences of PPH between service users and health care providers and where acceptable treatments and techniques in line with this frame of reference are available free at the point of use. These systems should be applied sensitively and competently by care providers who are skilled and trained in their appropriate use; full information is available to childbearing women about side-effects of drugs and treatments and their alleviation; and where access to treatments and uterotonic drugs is not likely to result in their use in non-indicated circumstances. specially where it is based on their individual needs, beliefs and values. However, there is variation within and between healthcare providers and women, across income settings, in their views as to how far these interventions should be used routinely. In all income settings, systems for PPH prevention are likely to be more successful locally where there is a common frame of reference for the causes and consequences of PPH between service users and health care providers and where acceptable treatments and techniques in line with this frame of reference are available free at the point of use. These systems should be applied sensitively and competently by care providers who are skilled and trained in their appropriate use; full information is available to childbearing women about side-effects of drugs and treatments and their alleviation; and where access to treatments and uterotonic drugs is not likely to result in their use in non-indicated circumstances.
*Influence of traditional beliefs and treatments [28–30,32–34]*			
*Knowledge of complications varies [28–30,32,33]*			
*Want more information about PPH [24,26,27,35,54]*			
*Resource constraints hinder care [27–29,31,33,34]*			
*Value shared decision making [25,26,35]*			
*Value woman-centred care [23–26,31,36,54]*			
*Value clinical competence [23,24,27,33]*	*Importance of woman-centred care*. *Importance of clinical competence*. *Disrespect and abuse by staff*. **Importance of shared decision-making.**	The value of competent caring.	
*Experience and fear of mistreatment [26,27,31,36,54]*			
*Fatigue and exhaustion after PPH [24,25,27,34,35]*			
*Separation anxiety after PPH [24–26,35]*			
*Feelings of disorientation during PPH [24,26,29]*			
*Long-term psychological impact of PPH [24–27,35]*			
Selective use of guidelines [37–40,45,49]			
Preference for expectant management [37–40,49]	Recognition of preventative action of uterotonics. Implementation concerns around use of uterotonics for PPH prevention. Uterotonic safety concerns.	Influence of uterotonics in PPH prevention.	
Policy concerns around use of misoprostol [42,53–57]			
Staff want more training on PPH management [29,30,38–40,43–45,47,48,51,53,54,56]			
Resource constraints hinder practice [30,40–49,51,53,55,57]			
Safety concerns (oxytocin) [38,41,47–49]			
Safety concerns (misoprostol) [41,42,52,53,55–57]			
Trust in task shifting [29,40,43,44,46,51,53,55,57]			
Perception and understanding of blood loss [29,30,40,50,54]	**Lack of resources** Influence of hierarchical structures in perpetuating poor practice. Trust in task shifting.	Organizational issues affect PPH prevention.	
Influence of traditional beliefs and treatments [29,30,43,44,47,48,50,51]			
Influence of community distribution programmes (misoprostol) [2,55–57]			
Recognition of benefits of uterotonics [40–42,44,48,51]			
Hierarchical systems hinder change [40,45,54]			
Value early attachment after PPH [37–39]			
Value informed decision making [39,46]			

Text in italics indicates findings from women

Text in bold indicates finding from both women and healthcare providers

### Theme 1: What PPH means to stakeholders: belief, knowledge, and understanding

This theme encompasses the way in which women and care providers think about blood loss after childbirth, and what it means in this context. It highlights a variety of traditional, personal and clinical beliefs relating to the nature of, and prevention of PPH. It also describes what service users and providers say about their need for information about PPH, in terms of knowledge from a woman’s perspective and additional training needs from a healthcare provider perspective.

#### Understanding of blood loss

For women living in a variety of predominantly rural settings in LMIC’s the release of 'dirty blood' after childbirth was perceived as a normal cleansing process and something that shouldn't be prevented. “*The blood that flows after childbirth is bad blood because for the whole pregnancy you are not menstruating and this is a long period*. *When that blood remains in your womb*, *you might get some complications*”. (20 year old woman, Uganda [29]). In communities where this view was prevalent it was held by both childbearing women and traditional birth attendants (TBA’s), who saw little reason to adopt preventative measures and sometimes regarded prevention of postpartum blood flow as a dangerous process. The distinction between what constituted normal blood loss and what might be considered dangerous was often blurred and relied on simplistic measures such as visual inspection of buckets, cloths and pads. However, these respondents also showed awareness of the physiological signs of extreme blood loss and shock: *The woman’s body becomes blue* (nila hoiya jay) *from continuous bleeding*. *The blood goes out in a continuous*, *swift flow or gush* (dhala dhala), *it overflows the place*. *Two or three jute-made bags* (chala-chula) *will not control or stop the blood*. (Postpartum woman, Bangladesh [30]).

#### Impact of PPH

Women in several high-income settings recounted their experience of having a PPH and their hope that their stories might raise awareness of the condition from a preventative perspective. They talked about overwhelming feelings of exhaustion and fatigue following the PPH as well as powerful feelings of disorientation that affected communication with healthcare staff during the PPH. They reported feeling anxious and frustrated at not being able to ‘bond’ with their baby immediately after birth and discussed the long-term psychological impact of having a traumatic experience, “*More (information) is needed about the emotional difficulties after experiencing such a traumatic birth*. *I wasn’t prepared for this and suffered as a result*” (Primiparous woman, 2 months postpartum, New Zealand [27])

#### Traditional means of preventing or treating PPH

In a number of different LMIC's women adopted a variety of traditional practices to prevent and treat PPH. In some contexts spiritual or supernatural forces were thought to be responsible, and prevention therefore depended on appeasing these forces: “*Dushi (evil spirit) turns on a pregnant woman if she stays outdoors in the early morning*, *in the evening*, *and at noon*, *ignoring rules*, *and heavy bleeding occurs in this case”* (Woman in Bangladesh [28]). Prevention of PPH also included mechanical techniques that recognised the need for compression: *‘Immediately after the expulsion of the placenta*, *I massage the woman’s abdomen*, *tie a cloth around the abdomen and advise her to lie on her tummy until the bleeding stops*.*’* (56 year old female TBA, South Africa [50]). In some contexts TBA’s used herbs and teas with uterotonic properties to prevent PPH. Though such treatments might actually have pharmacological justification, the practice was frowned upon by some healthcare professionals: “*We do not know what the side effects might be*. *The problem is that the matrone (TBA) does not even know about the right dosage*, *what may happen if it is too much…For example*, *she may tell the woman to boil a handful of the plant but the problem is that a handful for the matrone might be different from the handful of the woman*. *So*, *there might be a risk of overdose and I am sure that it provokes [retained placenta]*. (Obstetrician, Madagascar [47]).

#### Knowledge about PPH

Several studies conducted in LMIC’s showed that women were often knowledgeable about PPH. Younger women, in particular, acknowledged that it was a complication that could and should be prevented. Where explanations were given they were generally vivid and clear: “*This bleeding is so severe that a pad or cloth cannot hold it*. *The bleeding runs down the legs of the woman*. *At times it is like a running tap that the bleeding can fill up a bucket*. *Such bleeding may cause the death of the woman”* (Woman in rural Nigeria [33]).

#### Desire for more information

Although knowledge of PPH as a potential complication was fairly widespread, the desire for more information about all aspects of the condition was expressed by women in a number of settings, especially in high-income countries (HIC’s). Participants reflecting on their experiences of a PPH described poor communication by healthcare providers as a particularly frustrating aspect of their care and suggested information leaflets and/or relevant websites might address some of their concerns, especially if these were discussed with healthcare providers during antenatal appointments.

#### Desire for more staff training

Across a wide range of settings and contexts, healthcare providers identified knowledge gaps in their understanding of PPH management, expressed a lack of confidence in their ability to manage PPH and highlighted a lack of training opportunities to address these concerns, particularly in LMIC's. *“I have been working for over ten years and have had a refresher course only once*. *You can imagine*, *every time they (policy makers) talk about maternal death instead of giving health workers some more training on PPH…*.*”* (Medical assistant, Malawi [43]). In high-income settings the uncertainty around AMTSL practices caused confusion amongst health professionals and was usually attributed to lack of knowledge *“Even if it is stated in the (PPH) protocols*, *team members still doubt the content*, *which leads to tedious discussions*. *This is mainly caused by lack of knowledge*” (Junior doctor, The Netherlands [54]).

#### Provider preferences for PPH prevention

Some healthcare providers remained unconvinced about the benefits of using uterotonics (primarily oxytocin) to prevent PPH and ignored pertinent guideline recommendations to better align with their personal beliefs. These beliefs tended to be based on notions of normality and the understanding that a woman’s embodied experience would lead to the appropriate course of action, *“I don’t completely agree with having guidelines that must be strictly followed without thinking why; when you give it*, *why do you give it*? *So I choose the middle way*, *I mean if a normal primipara isn’t bleeding I write ‘Not given oxytocin’ in her notes*. *I see no reason to give it”* (Midwife, Sweden [38]).

Health professionals also expressed a preference for a 'hands off' approach to third stage management believing that this technique didn't add to women's pain and produced better PPH outcomes when birth was perceived to be normal. *“After the delivery I didn’t give oxytocin because she delivered normally*. *Only if she is bleeding*. *Because some of them they deliver normally*, *no problem*.*”* (Maternal and Child Health Aide, Sierra Leone [49]).

### Theme 2 –Organizational issues affect PPH prevention

This theme highlights some of the organizational issues that impede the effective management of PPH. Healthcare providers and, in some cases, women, identified resource limitations, hierarchical structures and a lack of trust in working relationships as potential barriers to PPH prevention.

#### Resource constraints

Staff shortages and lack of medicines or equipment were consistently highlighted by healthcare providers in a wide range of settings and contexts. Staff shortages were particularly acute in a number of LMIC’s and were blamed for maternal deaths, *“Another major cause of maternal death is lack of enough skilled workers in this midwifery practice”* (Nurse-midwife, Malawi [43]). Inconsistent or limited stock levels of oxytocin were widespread in a number of LMIC’s and meant that providers were sometimes forced into using EMTSL, even though they perceived this to be sub-optimal care, *“We give oxytocin if we have*, *but if we don’t have then we express normal procedures [expectant management]*.*”* (Maternal and Child Health Aide, Sierra Leone [49]). Limited supplies of oxytocin at health facilities sometimes had a knock-on effect for women because healthcare providers insisted on following AMTSL recommendations (out of fear of litigation) and asked women to buy oxytocin privately before coming to the health facility to give birth, *“It’s a challenge when you tell them go and buy this drug because the hospital can't provide it and they discover that it’s expensive*. *The hospital provides it sometimes*, *but sometimes it runs out*.*”* (Junior doctor, Uganda [45]).

#### Trust in Task Shifting

To address the relatively high maternal mortality rates in some LMIC’s, national and local governments have introduced policies to train cadres with lower levels of training (mainly community health workers and traditional birth attendants) on how to use uterotonics, largely misoprostol, to prevent PPH in community settings. In some contexts, especially where home births were commonplace, health professionals thought this was a good idea, *“I don’t differentiate between the health extension workers and the trained volunteers or the TBAs or anyone else*. *As long as they have received the proper training*, *they are more than capable of administering the drug”*. (Healthcare provider, Ethiopia [57]). However, in other contexts, healthcare providers felt that TBA's and community health workers might use uterotonics irresponsibly, *“You see they [TBA’s] know it as “Tha ein pwint say” and we are worried that they would use it before delivery thinking that it will enhance labour*. *So we emphasize in the training as “tha ein kyunte say” and we stress that it can only be given after the birth of the baby”* (Auxiliary midwife trainer, Myanmar [53]).

#### Influence of hierarchical structures in perpetuating poor practice

In a limited number of settings healthcare providers felt that their ability to influence change in the practice of AMTSL was hampered by existing hierarchical structures. A culture of repressive seniority coupled with a fear of repercussion prevented genuine dialogue that might improve clinical skills, *“We were told to do the counter traction this way (showing the right manoeuvre)*, *put pressure on the uterus upwards*, *and then start retracting the placenta*. *But when I came here*, *I realized some of my colleagues do it the other way round*, *they hold the uterus this way (showing the opposite grip) and they pull*. *But they are my seniors so if I say “Don’t do it this way or that way”*, *they would feel that I was looking down at them”* (Labour ward midwife, Ghana [40]).

### Theme 3—The value of competent caring

This theme discusses some of the clinical skills and personal qualities valued by women (and healthcare providers) during their encounters with staff practicing AMTSL. It also highlights some disrespectful practices that women experience in certain low-income contexts.

#### Importance of clinical competence

Women in a limited number of high-income settings expressed a sense of reassurance when staff were perceived to be clinically competent. They felt safe when health professionals gave injections efficiently and painlessly and remained calm when AMTSL procedures were carried out with minimum fuss. By contrast, for a small number of women in rural Nigeria the perceived incompetence of staff at the local health facility deterred women from attending for potential childbirth complications. For these women the local TBA’s were regarded as more competent in PPH management. The author of this study notes that, “*nurses and trained midwives are both ill-experienced and untrained to undertake simple methods of controlling haemorrhage*, *or handling other complications”* [33].

#### Importance of woman-centred care

As with most other aspects of maternity care the importance of kind, empathic, personal care was highly valued by women in a variety of settings. Being acknowledged and reassured were key features of this approach, *“the care up to that point had been so good and everybody had been*, *um*, *so supportive*, *not that I’d expected otherwise but I was quite*, *um*, *pleased with the time they took just to reassure me and … that sort of personal touch*, *do you know what I mean*, *was really nice”* (Post-natal woman, UK [24]). For some women the opportunity to engage with staff on a personal level demonstrated a woman-centred approach, while for others the simple act of receiving attention was perceived favourably, *"The treatment was good*, *I liked it a lot*. *The nurse at sixth floor gave me a lot of attention and she continued in the delivery room"* (Multiparous woman, Angola [31]).

#### Importance of shared-decision making

Women and healthcare providers both discussed shared decision-making when talking about their experiences of AMTSL. Women expressed feelings of frustration and anxiety when decisions were taken without their involvement and feelings of control and empowerment when they were actively engaged in decisions relating to third stage practices, “*I was very appreciative of the fact that [my midwives] gave me the info that I could handle for the situation that I was in*. *They didn’t hide anything*, *and they certainly answered every question that I had…so I was fine*. *I was calm”* (Multiparous woman, Canada [25]). Healthcare providers in a limited number of settings also recognized the benefits of shared decision making and used terms like ‘option’ and ‘choice’ rather than ‘recommendation’ when discussing clinical practices in the third stage of labour [39].

#### Disrespect and abuse by staff

In a relatively small number of diverse settings (including HIC’s and LMIC’s) women experienced disrespectful, rude and sometimes abusive behaviour by healthcare providers. In some LMIC contexts, there was an apparent expectation that women should ‘behave’ during all stages of childbirth and, if they failed to do so, they would be punished by healthcare providers, *"I was well treated because I did not cause a scandal*. *The others who were shouting and crying were badly treated"* (Multiparous woman, Angola [31]). In other low income contexts women experiencing a secondary PPH were punished, admonished, mistreated or even refused care because they had given birth at home rather than in a health facility, “*No we did not go to the hospital [for treatment] because if they notice that you delivered from the village … she [the nurse] can easily beat you*. *So it is better I go to the old lady in the village because I know she will treat me well”*. (Postpartum woman, Uganda [36]).

### Theme 4 –Influence of uterotonics in PPH prevention

This theme discusses some of the issues surrounding the use of uterotonics to prevent PPH. Whilst acceptance of the effectiveness of uterotonics (generally oxytocin and misoprostol) was fairly widespread, healthcare providers raised concerns about a number of issues relating to safety, logistics, implementation strategies and the influence of non-governmental organizations.

#### Recognition of the benefits of using uterotonics for PPH prevention

Healthcare providers in a wide variety of settings and contexts acknowledged that both oxytocin and misoprostol were effective agents in strategies to reduce PPH, *“One of the protocols is that when the woman has delivered we give oxytocin*. . . . *All of this management is an attempt to prevent PPH*.*”* (Clinical Health Officer, Sierra Leone [49]). A number of healthcare providers had been involved with advance community distribution programmes and felt that misoprostol was a more practical and acceptable solution to PPH prevention in these contexts, *“The women are grateful because they are afraid of bleeding*. *But with this tablet (misoprostol) they become more at ease*. *She was bleeding when she delivered her first baby*, *but the second one*, *she participated in the programme*, *got the medicine and there was no bleeding”* (TBA, West Java [52])

#### Safety concerns about uterotonic use

Healthcare providers in a number of LMIC’s highlighted specific safety concerns around the use of misoprostol, particularly in community settings. Although side effects were identified (especially shivering), they were usually considered to be transient and reasonably acceptable to women. Concerns were more commonly raised by providers when discussing advanced community distribution programmes and the potential for mis-use of the drug, “*No one should overestimate misoprostol because many things can cause bleeding*. *What will happen if the drug is given when the woman is still in labor*? *Or if there is another baby (twin)*? *Or perhaps it may be used unsafely for other purposes*… *A woman can die”*. (Healthcare provider, Ethiopia [57]). Some healthcare providers also expressed concerns about the administration of oxytocin and the perception that it might cause retained placenta “*Some women are in a lot of pain and it’s my impression that we’ve had more retentions since we started giving those 10 units of oxytocin*. *The placenta gets trapped in the cervix”* (Experienced midwife, Sweden [38]).

#### Implementation concerns

In a number of LMIC’s healthcare providers had reservations about advance distribution programmes for misoprostol. For some policy level administrators, there were unanswered questions about the effectiveness of these programmes, “*There is a general policy that guides us*. *But how should misoprostol be given*? *Who should it be given to*? *How it should be implemented*? *A document that includes all of these things should be compiled*. *I think there is much that has not been done”*. (Healthcare provider, Ethiopia [57]). For others, there were concerns that advance distribution programmes (for misoprostol) could undermine efforts to strengthen safe delivery services or encourage unregulated providers to distribute misoprostol irresponsibly, *“There needs to be controls otherwise sellers will advertise and people will misuse [the drug]”* (Healthcare provider, Laos [56]). In a smaller number of LMIC’s providers also voiced concerns about implementation strategies to reduce PPH using oxytocin, though these were largely limited to reservations about the cold-chain storage of the drug, “….*For oxytocin you have to move around with a refrigerator to keep the cold chain*. *In rural communities this is not possible”*. (Healthcare provider Ethiopia [57]).

#### Line of argument synthesis

Women who give birth and healthcare providers recognise that severe, uncontrollable blood loss in the peri- and postpartum period is life threatening. Based on their beliefs and experiences, birth attendants can identify women at risk of PPH, and most are willing to use specific skills and techniques (and, where available, treatments and uterotonic drugs) to reduce the risk. Women generally appreciate this, especially where it is done on the basis of their individual needs, beliefs and values. However, there is variation within and between healthcare providers and women, across income settings, in their views as to how far these interventions should be used routinely. In all income settings, systems for PPH prevention are likely to be more successful locally where there is a common frame of reference for the causes and consequences of PPH between service users and healthcare providers and acceptable treatments and techniques in line with this frame of reference are available free at the point of use. These systems should be applied sensitively and competently by care providers who are skilled and trained in their appropriate use; full information is available to childbearing women about side-effects of drugs and treatments and their alleviation; and where access to treatments and uterotonic drugs is not likely to result in their use in non-indicated circumstances.

## Discussion

Our findings suggest that women and healthcare providers are aware of the potentially catastrophic consequences of uncontrollable postpartum bleeding, and that many communities and health care systems recognise warning signs for it. These range from physiological and psychological cues, to an assessment of the quantity of blood lost, by various means, most of which are very basic, even in high-income settings. However, all groups recognise that some blood loss is normal, and even that it might be essential, for example, to prevent the retention of ‘dirty blood’. It is possible that this term is used colloquially because of the association between retained products of labour and intrauterine infection, and this would indicate empirical knowledge of the consequence of apparently low blood loss in some women who later become very ill.

Whilst our findings indicate that women were generally aware of the consequences of a severe PPH, in some contexts the reliance on traditional healers to manage potential childbirth complications appeared to be born out of economic necessity and/or localized cultural belief systems. Some of the traditional preventative treatments advocated by TBAs or untrained care providers reflect the general physiological principles in this area (for example, compression and the use of herbs and agents with uterotonic properties). However, some of the more supernatural beliefs, e.g. protection against evil spirits, conflict with Westernized understandings of PPH and remain a challenge in some LMICs [58, 59, 60]. Formal education programmes designed to increase awareness of the potential dangers of PPH may improve understanding in these contexts [61], although recent research conducted in rural contexts in Kenya and Malawi suggests community-led women’s groups may be more beneficial in raising awareness of maternal complications (including PPH) in these communities [62, 63].

Apart from survival, other outcomes that matter to women in connection with prevention of PPH include avoidance of the profound fatigue, psychological distress, and the longer-term emotional consequences of the trauma of severe blood loss. The emotional impact of a PPH is sometimes overlooked in the literature but research suggests that, for some women, the repercussions can be severe and associated with long-term mental health problems including post-traumatic stress disorder [64]. However, there seems to be a general view that, if treatments are needed to prevent these adverse outcomes, they will be acceptable if both the need for the treatment and the nature and alleviation of potential side effects are explained, and if the treatment is provided by competent, caring staff. These findings are in accord with other reviews of what matters to women in maternity care provision [65,66].

Provider views on the benefits of using clinical interventions to prevent PPH (active management) were generally favourable but some healthcare providers were more circumspect in their approach, and didn’t intervene if they felt labour and birth had proceeded ‘normally’ (expectant management). Reasons for adopting an expectant approach are unclear but may be related to providers personal beliefs around ‘normality’ or a desire to respect women’s preferences, particularly amongst midwives in high-income settings where all of the resources to manage a severe PPH are readily accessible and available [67]

From an organizational perspective our findings indicate that where healthcare providers are adequately trained in PPH prevention and management and have access to appropriate resources, both in terms of staff and medicines, they are reasonably confident in their practice. However, in situations where training opportunities are limited, where guidelines are unclear or outdated and where hierarchical structures inhibit staff development, providers may feel undervalued or unsure of their PPH management skills. These findings are in accord with a recent study exploring AMTSL practices in 7 LMICs (from Asia, Africa and Central America) where the authors found substantial variations in the correct use of uterotonic drugs to prevent PPH and attributed these inconsistencies to inadequate training, outdated or contradictory guidelines and/or poor organizational standards [68].

In LMICs where resources may be limited, the use of task-shifting to address staff shortages is sometimes seen as a solution and, although our findings generally support this practice, some healthcare providers had reservations about the ability of lower level cadres to provide accurate information to women, or use uterotonics safely. Findings from a qualitative review looking at barriers and facilitators to the implementation of lay health worker programmes to improve access to maternal health identified trust as a potential concern amongst healthcare providers, but the same review highlights health system support, adequate training and community endorsement as potential facilitators to effective implementation [69]. In situations where healthcare providers may be thinking about using task-shifting to address PPH prevention and management, these implementation issues need to be considered.

Where uterotonic agents were available (such as oxytocin or misoprostol) they seemed to be generally acceptable to women. These views are supported by a number of other studies, particularly in relation to advance community distribution programmes for misoprostol, where women either safely self-administered the drug at home or were given the correct dose at the appropriate time by community health workers or TBA’s [70,71,72]. In this review, some healthcare providers were cautious about these programmes because of concerns relating to the effectiveness of misoprostol, it’s side-effect profile and the potential for mis-use in unregulated settings. These observations reflect a wider debate about the benefit/harm balance of community distribution programmes for misoprostol in LMICs where advocates emphasize the value of the drug in reducing maternal mortality whilst doubters highlight the ethical principle of non-maleficence [73]. From a guideline perspective, WHO recommends oxytocin (in injectable form) as the uterotonic of choice to prevent PPH, and recommend other injectable uterotonics or oral misoprostol as alternatives when oxytocin is unavailable [13]. In reality, the practicality of using a widely available, cost-effective, heat-stable drug in situations where women may not want or be able to attend health facilities to receive oxytocin may lead to a preference for misoprostol. Evidence from a number of studies commissioned by aid agencies working in areas with relatively high levels of maternal mortality suggest that this is indeed the case [74,75,76].

In terms of strengths and limitations, the number of papers included in this review and the geographical spread of the populations they represent implies that the findings are likely to be transferable. Twenty-two of the 35 included studies were conducted in LMIC’s with the majority being published in the last 10 years. This suggests that the data is current and of particular relevance to the locations in the world where the prevention of PPH is most important. Although our findings address the broad nature of the review question and are comprehensive in nature, we were unable to locate many studies that directly reflected the views and experiences of women and healthcare providers regarding the prevention of PPH. This was particularly evident for women where the studies were largely framed around experiences of PPH or the understanding and beliefs associated with postpartum bleeding. Going forward, it would be useful if future qualitative studies sought to address this deficit by exploring preventative approaches to PPH more directly. Women and healthcare providers are likely to offer valuable insights into the acceptability, feasibility and implementation of PPH prevention practices that lie beyond the scope of effectiveness studies.

In summary, our findings suggest that women and healthcare providers expect some bleeding after birth, and, indeed, may even see it as physiologically beneficial for the woman. However, the review also shows that many women understand the nature and risks of excessive bleeding, and, generally, that they find a range of traditional and pharmacological preventative treatments to be acceptable in preventing this. In some populations, there is resistance among both lay and professional healthcare providers to adopt routine preventative approaches, in favour of effective and rapid treatment where an individual woman is bleeding heavily and/or showing signs of deterioration. This suggests that guidelines for routine active management of third stage of labour may be less acceptable in such contexts. Furthermore, in low-income settings, a lack of resources including adequately trained staff, outdated local policies and the availability of relevant pharmaceutical agents inhibits guideline implementation. Some of these issues may be resolved by task shifting practices and/or advance community distribution programmes (for misoprostol) but these require careful planning and effective management. In all settings, as in all other reviews of what matters to women in maternity care, optimising the opportunity to have a positive maternity care experience seems to be essential for the future wellbeing of the woman and her on-going relationship with her baby. In terms of prevention of PPH, as for general antenatal and intrapartum care, this includes support, information, and clinical treatments all provided by competent, caring staff, with the resources to supply what is needed, to all those that need it, free at the point of care [65,66].

## Conclusion

Our review highlights inconsistencies in the understanding of post-partum blood loss amongst women and healthcare providers. Policies designed to prevent PPH need to establish a coherent understanding of this issue amongst relevant stakeholders and find agreement on the causes and consequences of severe postpartum blood loss. Preventative strategies also need to pay attention to some of the outcomes that are of value to women and healthcare providers. Interventions and programmes focused on PPH prevention are more likely to be successful if they are acceptable to stakeholders, feasible to implement and sufficiently resourced to ensure any potential benefits are optimised across all income settings.

## Supporting information

S1 Table PRISMA checklist. (DOCX)

S1 Appendix Search strategy example. (TIF)

S2 Appendix Study selection and CERQual grading. (XLSX)
