# Supplementary figures and images for: What matters to women and healthcare providers in relation to interventions for the prevention of postpartum haemorrhage: A qualitative systematic review
Source: PLoS One. 2019 May 8;14(5):e0215919. doi: 10.1371/journal.pone.0215919 (PMC6505942; doi:10.1371/journal.pone.0215919)

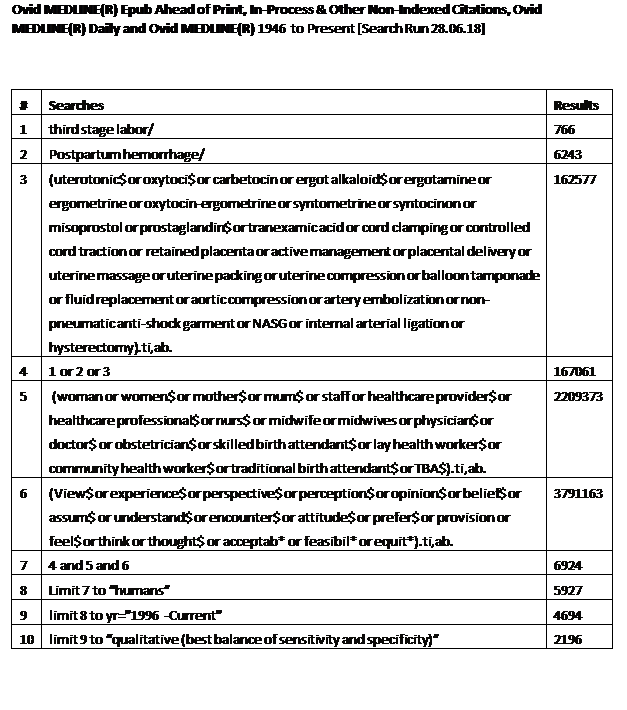

Supplement: S1 Appendix — (TIF) [file pone.0215919.s002.tif]
